# Computational Discovery of Angiotensin-(1-7)-like Peptides Targeting the MAS Receptor from the Genomic Dark Matter of *Saccharomyces cerevisiae*

**DOI:** 10.7150/ijms.135093

**Published:** 2026-07-01

**Authors:** Pattayampadam Ramakrishnan Shidhi, Aiswarya Jayaprakash, Navya Raj, Peramaiyan Rajendran, Rebai Ben Ammar, Biju Vadakkemukadiyil Chellapan

**Affiliations:** 1 Department of Zoology, University of Kerala, Karyavattom, Trivandrum, Kerala, 695581, India.; 2 Department of Computational Biology and Bioinformatics, University of Kerala, Karyavattom, Trivandrum, Kerala, 695581, India.; 3 Department of Health Informatics, College of Health Sciences, Saudi Electronic University, Kingdom of Saudi Arabia.; 4 Department of Biological Sciences, College of Science, King Faisal University, Al-Ahsa, 31982, Saudi Arabia.

**Keywords:** dark matter, *Saccharomyces cerevisiae*, peptides, therapeutic peptides, non-coding DNA

## Abstract

Non-coding regions of eukaryotic genomes, once considered transcriptionally inert, are increasingly recognized as potential sources of bioactive peptides. Here, we mined the intergenic “genomic dark matter” of Saccharomyces cerevisiae to identify peptide ligands targeting the angiotensin-(1-7)/Mas receptor axis. Intergenic sequences were computationally translated in all reading frames and screened for short peptides with sequence and structural similarity to angiotensin-(1-7), a key modulator of the renin-angiotensin system. Two candidates showing 72-86% similarity were identified and structurally modeled. A homology-independent model of the human Mas receptor was generated, followed by molecular docking and 100-ns membrane-embedded molecular dynamics simulations to assess binding modes and complex stability. Both peptides adopted angiotensin-(1-7)-like conformations and engaged conserved receptor residues involved in ligand recognition. Docking and dynamics analyses indicated stable binding and sustained receptor engagement, with one peptide exhibiting enhanced interaction density and conformational stability relative to the native ligand. Overall, our results support the concept that non-coding genomic regions can encode functional peptide ligands and provide a scalable in silico framework for discovering peptide modulators of clinically relevant G protein-coupled receptors (GPCRs).

## 1. Introduction

Only a small fraction of eukaryotic genomes encodes annotated proteins, yet the remaining “dark” regions are increasingly recognized as a rich source of bioactive peptides. Indeed, roughly 1% of the human genome is protein-coding, with the vast majority transcribed into noncoding RNAs 1. Recent ribosome profiling and proteogenomic studies have uncovered thousands of previously unannotated open reading frames (ORFs) embedded in these noncoding loci 2,3. Many of these cryptic microproteins - often under 100 amino acids - are actively translated and can carry out diverse regulatory functions. For example, lncRNA-encoded peptides have been shown to modulate muscle physiology, metabolism, immune signaling and more 4,5. A pan-eukaryotic analysis found that hundreds of stable peptides arise from 5′- or 3′-untranslated regions and lncRNAs (including in yeast), indicating that even well-studied genomes harbor a substantial hidden proteome 6. Collectively, these findings suggest that mining non-canonical genomic regions may yield novel bioactive sequences.

Intergenic regions have traditionally been considered non-coding; however, recent ribosome profiling and proteogenomic studies have shown that non-canonical ORFs located outside annotated protein-coding genes can be transcribed and translated into short peptides or microproteins 7,8. Some of these peptides have been reported to participate in regulatory, metabolic, and signaling processes, indicating that unannotated genomic regions may represent an underexplored reservoir of bioactive molecules. Based on this emerging evidence, we investigated whether intergenic regions of Saccharomyces cerevisiae could encode short peptide sequences with similarity to known bioactive ligands such as angiotensin-(1-7) (Ang-(1-7)).

Ang-(1-7)) is a naturally occurring heptapeptide hormone in the renin-angiotensin system (RAS) that counterbalances classical angiotensin II signaling 9,10. Ang-(1-7) is generated by ACE2-mediated cleavage of Ang II and primarily signals through the G-protein-coupled Mas receptor 11. Early work showed that Ang-(1-7) causes vasodilation and opposes Ang II-induced vasoconstriction, and subsequent studies have documented potent anti-inflammatory and anti-fibrotic effects through Mas activation. For instance, in animal models of sepsis or injury, Ang-(1-7) treatment markedly reduces proinflammatory cytokines (e.g., TNF-α, IL-6) while increasing anti-inflammatory IL-10, promoting M2 macrophage polarization and tissue protection 12,13. Notably, these beneficial effects are lost in Mas-knockout systems, underscoring that the Ang-(1-7)/Mas axis is an endogenous protective pathway 14. The therapeutic potential of this axis has attracted attention in cardiovascular, pulmonary and metabolic diseases, where Ang-(1-7) or its analogues are being explored as drug candidates 15.

Following recent advances in peptide drug discovery, driven by high-throughput screening, bioinformatic design, and chemical synthesis, computational approaches enable systematic mining of non-coding DNA for novel peptide ligands with therapeutic potential. In the present study, we sought to harness these emerging paradigms by mining the non-coding genome of *Saccharomyces cerevisiae* for peptides with structural and functional similarity to Ang-(1-7). The compact and extensively annotated genome of *S. cerevisiae*, coupled with its known transcriptional activity in intergenic regions, presents an ideal model system for this investigation. We employed a computational pipeline to translate intergenic sequences, model the resulting peptides, and assess their structural compatibility with Ang-(1-7). To evaluate their biological relevance, we modeled the MAS receptor, performed peptide-receptor docking, and simulated the dynamics of the resulting complexes. This integrative approach aims to uncover bioactive molecules encoded within genomic dark matter and expand the repertoire of peptide-based modulators of the RAS pathway.

## 2. Materials and Methods

### 2.1. Identification of Ang-(1-7)-like peptides from intergenic regions

Intergenic sequences of *S. cerevisiae* were downloaded from the reference Saccharomyces Genome Database (SGD) 16. A total of 6,584 annotated intergenic sequences were included in the analysis. To identify potential peptide-coding regions within these non-coding sequences, each intergenic sequence was translated in all six possible reading frames using EMBOSS Transeq. The resulting translated peptide dataset was used as the initial pool of putative dark peptides.

The translated peptide pool was screened for short peptide fragments showing similarity to Ang-(1-7). Candidate peptides were first filtered based on length comparable to the native heptapeptide ligand. The remaining peptides were then evaluated for sequence similarity to Ang-(1-7) using local sequence-alignment-based screening. Peptides were prioritized according to percentage sequence similarity, conservation of key physicochemical features, including charged, aromatic, and hydrophobic residues, and absence of overlap with annotated protein-coding regions.

Because six-frame translation of intergenic regions generated a large number of putative peptide fragments, a stepwise prioritization strategy was applied to identify candidates most suitable for downstream analysis. Peptides with low similarity to Ang-(1-7), poor physicochemical correspondence, or unsuitable sequence features for reliable structural comparison were excluded. Only the highest-priority candidates satisfying the combined criteria of sequence similarity, ligand-like length, physicochemical compatibility, and suitability for structural modeling were selected for conformational comparison with Ang-(1-7), molecular docking with the MAS receptor, and molecular dynamics (MD) simulation.

### 2.2. Modeling the structure of MAS-receptor

The MAS receptor sequence (UniProt ID: P04201) was retrieved in FASTA format from the UniProt database. Homologous proteins were identified by querying the Protein Data Bank (PDB) using BLASTp 17, and template structures were selected based on sequence similarity and E-value. As the highest similarity was only 28%, the three-dimensional structure of the MAS receptor was predicted using the I-TASSER server 18, which integrates *ab initio* modeling with threading approaches. Structural validation of the predicted model was performed using SAVES and the WHAT_IF server 19 to assess stereochemical quality, planarity, and side-chain accessibility. Residues falling in disallowed regions of the Ramachandran plot were refined using ModLoop 20. Superimposition of I-TASSER-generated models was carried out in UCSF Chimera 21 to evaluate structural consistency, while secondary structure topology was further analyzed with PDBsum 22.

### 2.3. Prediction of transmembrane region and active site

The transmembrane region of the MAS receptor was predicted using Discovery studio 2019. The active site present in the MAS receptor was identified through literature study and it was further validated using SiteMap tool in Schrödinger Software suite 23, 24.

### 2.4. Modeling and structure similarity of peptides

The 3D structure of Ang-(1-7) (PBD Id: 2JP8) peptide was retrieved from PDB. I-TASSER server was used to model the 3D structure of dark peptides. To predict the structural similarity, superimposition of dark peptides and Ang-(1-7) peptides was performed using the tool Superpose 26.

### 2.5. Molecular docking

Docking studies were performed with the MAS receptor to predict the functionality of Ang-(1-7) and dark peptides using the ZDOCK protocol 27-29 in Discovery studio 2019. ZDOCK a rigid-body protein-protein docking algorithm based on the Fast Fourier Transform correlation technique was used for docking studies. The ZDOCK results are refined by a program called RDOCK, a CHARMm - based energy minimization procedure for refining and scoring docked poses using energy scoring functions. The protein and peptide were prepared to correct missing residues or atoms and to remove the presence of any heteroatoms. All the structures were 'energy minimized'. The active site residues were selected in the prepared protein and docked against the peptides. The clustering parameter groups: Root Mean Square Deviation (RMSD) cutoff and Interface Cutoff was set to 6.0 and 9.0 respectively. The best docked poses were identified and refined through Zdock score, Zrank score, bonded and nonbonded interactions. Initially, protein-protein docking was performed with MAS receptor and Ang-(1-7) then with the dark peptides. The interacting residues for Ang-(1-7) and dark peptides were identified using Discovery studio. A comparative study was done with the docking and interaction results obtained for Ang-(1-7) and dark peptides to predict the binding affinity of Ang-(1-7) and dark peptides with the receptor MAS.

### 2.6. Molecular dynamic Simulation of docked complex

Molecular Dynamic simulation of docked complex was performed using GROMACS version 5.1.2. The membrane protein ligand complex of three docked complexes (Complex I - MAS + Ang-(1-7), Complex II-MAS + dark peptide 1, Complex III - MAS + dark peptide 2) with the simulation system was generated using CHARMM_GUI Membrane Builder using CHARMM 36 force field for energy calculations. The protein was embedded in two layers of POPE membrane with around 190 lipid molecules above and below the docked complex. The embedded protein ligand complexes in lipid bilayer are centered in a rectangular box filled with TIP3P water molecules. To achieve electro neutrality 7, 8, and 9 Cl- ions were added to each system of protein-ligand complexes to achieve electroneutrality. The simulations were carried out for 100 ns at 310.15 K and pH 7.0. The 100 ns timescale was chosen as a practical balance between computational cost and the need to observe equilibration, sustained receptor-peptide contacts, and backbone-level stability in a membrane-embedded GPCR system. Conformational stability and dynamic behavior were quantitatively assessed using root mean square deviation (RMSD), root mean square fluctuation (RMSF), radius of gyration (Rg), and hydrogen-bond analyses. These parameters were used to evaluate global structural deviation, residue-level flexibility, receptor compactness, and persistence of receptor-ligand interactions during the simulation.

## 3. Results

### 3.1. Identification, modeling, and structural comparison of dark peptides

**Figure 1:**
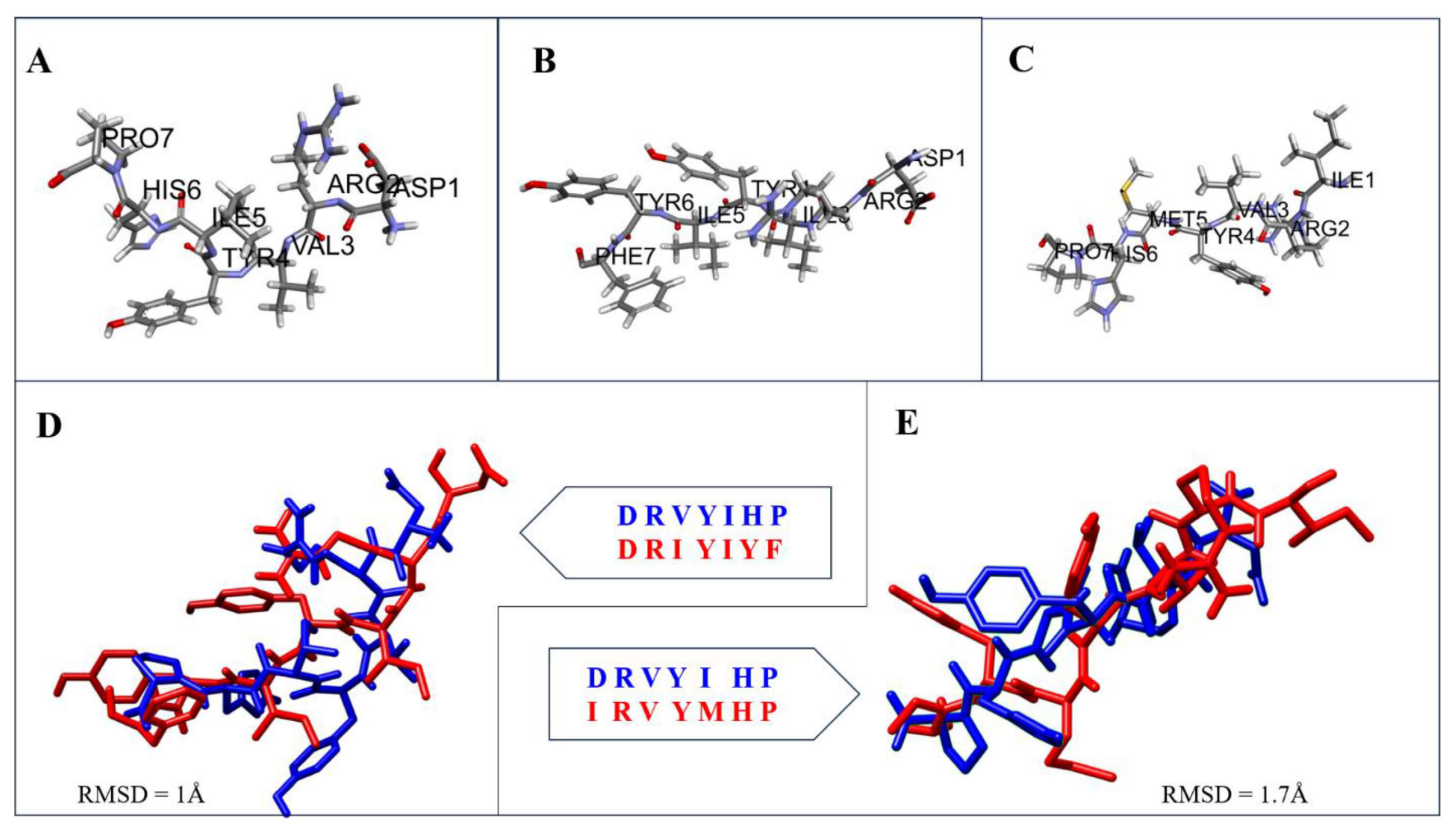
** Structural comparison of Ang-(1-7) and intergenic-region-derived peptides from *Saccharomyces cerevisiae*.** (A) Three-dimensional structure of Ang-(1-7). (B-C) Predicted three-dimensional structures of dark peptide 1 and dark peptide 2 generated using I-TASSER. (D-E) Structural superimposition of Ang-(1-7) with dark peptide 1 and dark peptide 2, respectively, showing close conformational overlap. The calculated RMSD values were **1.67 Å** for dark peptide 1 and **1.78 Å** for dark peptide 2 relative to Ang-(1-7), indicating structural similarity to the native peptide ligand.

To explore the potential of *Saccharomyces cerevisiae* intergenic regions to encode bioactive molecules, a total of 6,584 non-coding sequences were retrieved from the SGD and computationally translated in all six reading frames using Transeq (EMBL-EBI). The resulting peptide library was screened using BLASTX to identify sequences exhibiting similarity to known therapeutic peptides. Two short peptides translated from intergenic regions showed notable sequence similarity to the endogenous heptapeptide Ang-(1-7) (DRVYIHP), a critical modulator of the renin-angiotensin system (Figure 1A). These sequences were designated dark peptide 1 (DRIYIYF) and dark peptide 2 (IRVYMHP), displayed 72% and 86% sequence similarity, respectively, to Ang-(1-7), suggesting potential structural and functional mimicry.

The structural properties of the dark peptides were predicted using the I-TASSER platform. Both models yielded acceptable confidence scores (C-score = 0.36 and 0.58 for dark peptides 1 and 2, respectively), indicating reliable fold prediction (Figure 1B-C). The modeled peptides were subsequently superimposed with the experimental structure of Ang-(1-7) (PDB ID: 2JP8) to assess conformational similarity (Figure 1D-E). The calculated RMSD values were 1.67 Å for dark peptide 1 and 1.78 Å for dark peptide 2 (all-atom comparison), indicating a high degree of structural conservation with Ang-(1-7) (Figure 1). Moreover, the overlapping backbone alignment and preservation of key side-chain orientations further support that these dark peptides could mimic the spatial configuration of the native ligand.

### 3.2. Modeling, validation, and active-site characterization of the MAS receptor

The MAS receptor is a GPCR belonging to the rhodopsin-like class A family and serves as the principal binding site for Ang-(1-7) within the renin-angiotensin system. Upon ligand binding, it activates downstream Gq/PLC signaling pathways that mediate vasodilation, antifibrotic, and cardioprotective effects. Given the absence of an experimentally resolved structure for human MAS, *de novo* and template-based modeling approaches were employed to predict its three-dimensional conformation and to define the potential ligand-binding pocket.

The MAS receptor sequence (UniProt ID: P04201) was subjected to homology modeling using I-TASSER, as no experimentally resolved template with greater than 40% sequence identity was available. Among the five models predicted by I-TASSER, the structure with the highest confidence score (C-score = -0.28; expected TM-score = 0.68 ± 0.12; expected RMSD = 7.0 ± 4.1 Å) was selected. The top threading alignment utilized the human angiotensin receptor (PDB ID: 4YAY) as the primary template. A TM-score above 0.5 indicates a similar SCOP/CATH fold; notably, the human delta opioid 7TM receptor (PDB ID: 4N6H) showed a TM-score of 0.881, confirming high structural fold similarity to MAS. Functional annotation further suggested homology with the cholesterol-bound form of the human β₂-adrenergic receptor (PDB ID: 3D4S), indicating a comparable GPCR topology.

Structural validation demonstrated the reliability of the predicted model. The Ramachandran plot showed 83.8% of residues in the most favored regions (Figure S1), and the model achieved an overall quality factor of 88.64. Approximately 39.4% of residues exhibited a 3D-1D score >0.2, indicating good local geometry, while a few loop residues (1-11, 158-172, 278-298, and 312-325) showed scores below 0.0, reflecting minor orientation discrepancies. Planarity and accessibility analyses identified deviations in Glu322 and Asn317, whereas residues such as Met1, Arg21, Tyr95, His222, and Ser285 displayed high solvent accessibility, suggesting potential involvement in surface interactions.

**Figure 2:**
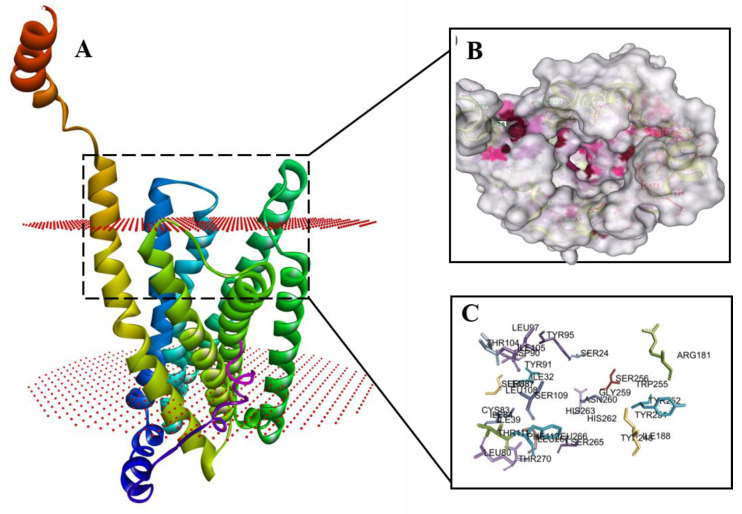
** Predicted structure and ligand-binding pocket of the human MAS receptor.** (A) Ribbon representation of the modeled MAS receptor showing the characteristic seven-transmembrane helical architecture of a class A GPCR. (B) Surface representation of the predicted extracellular ligand-binding pocket identified using SiteMap. (C) Key residues forming the putative Ang-(1-7)-binding region of the MAS receptor.

Topological analysis in PDBsum revealed that the modeled MAS receptor contains 11 α-helices, including seven transmembrane segments, 17 helix-helix interactions, and 24 β-turns, characteristic of class A G-protein-coupled receptors (GPCRs). Orientation assessment using the PPM server confirmed an extracellular N-terminus and a cytoplasmic C-terminus, characteristic of GPCRs. Analysis of TM helices and surface topology indicated the formation of a central pore-like structure across the membrane (Figure 2A).

Potential ligand-binding residues were identified through literature mining and validated using Schrödinger SiteMap (Figure 2B-C). A conserved extracellular pocket composed of Ile84, Ile86, Tyr95, Leu266, and Asn272 was defined as the primary binding region, consistent with previously characterized MAS receptor ligand-interaction sites (Figure 2C). Among these, Tyr95 and Asn272 occupy the extracellular loop interface critical for Ang-(1-7) recognition and were therefore selected as the principal docking interface for subsequent computational analyses.

### 3.3. Docking and interaction studies

To elucidate the molecular recognition determinants of MAS receptor activation and to evaluate the ability of the newly designed dark-genome peptides to mimic Ang-(1-7), molecular docking was performed using the refined MAS receptor model. Ang-(1-7), dark peptide1, and dark peptide2 were docked into the orthosteric binding groove, and the resulting complexes were analyzed for residue-specific interactions, hydrogen-bonding patterns, and aromatic/hydrophobic contacts.

**Figure 3:**
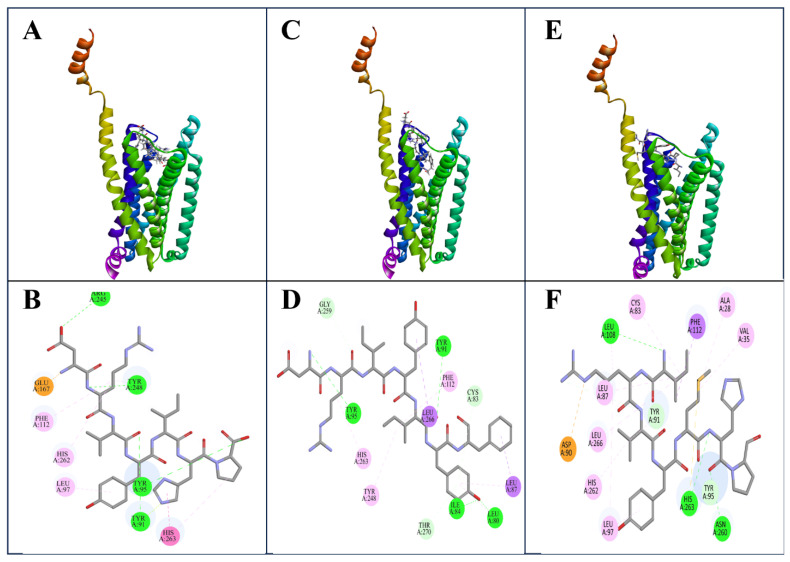
** Docking poses and interaction profiles of Ang-(1-7) and intergenic-region-derived peptides bound to the MAS receptor**. (A) Docked pose of Ang-(1-7) within the predicted MAS receptor binding pocket. (B) Two-dimensional interaction diagram of the Ang-(1-7)-MAS receptor complex. (C) Docked pose of dark peptide 1 within the MAS receptor binding pocket. (D) Two-dimensional interaction diagram of the dark peptide 1-MAS receptor complex. (E) Docked pose of dark peptide 2 within the MAS receptor binding pocket. (F) Two-dimensional interaction diagram of the dark peptide 2-MAS receptor complex. Interaction legend: Green = hydrogen bond; light green = carbon hydrogen bond; orange = salt-bridge/charge interaction; pink = π-π or π-alkyl interaction; purple = alkyl contact; yellow = π-sulfur interaction

Molecular docking revealed distinct but partially overlapping interaction patterns for Ang-(1-7), dark peptide1, and dark peptide2 within the orthosteric binding site of the MAS receptor (Table 1). The reference Ang-(1-7) complex displayed the characteristic MAS interaction signature, forming a strong salt bridge with GLU167 and a stabilizing hydrogen bond with ARG245, which together anchor the N-terminal portion of the peptide (Figure 3A & 3B). A dense network of conventional hydrogen bonds with TYR91, TYR95, and TYR248 stabilized the core of the ligand, while aromatic residues PHE112, HIS262, HIS263, and LEU97 contributed π-π and π-alkyl interactions that supported hydrophobic packing around the C-terminal region (Figure 3B). These features represent the canonical pharmacophore of Ang-(1-7) recognition (Table 1).

Docking of dark peptide2 produced a pose that overlapped closely with the native ligand but exhibited a far richer interaction profile (Figure 3C). Dark peptide2 preserved the essential TYR91 and TYR95 hydrogen bonds and formed new polar contacts with ASN260 and HIS263, the latter shifting from a hydrophobic π interaction (seen in Ang-(1-7)) to a stronger hydrogen bond (Figure 3D, Table 1). Instead of engaging GLU167 and ARG245, dark peptide2 established an alternative attractive charged interaction with ASP90, functioning as a substitute electrostatic anchor. Notably, a unique π-sulfur interaction with CYS83 and an expanded hydrophobic network involving LEU87, LEU97, LEU108, LEU266, VAL35, and ALA28 provided extensive stabilization, suggesting that dark peptide2 may bind with higher affinity and deeper insertion into the receptor pocket compared to Ang-(1-7) (Figure 3D, Table 1).

Dark peptide1 also adopted a pose similar to that of Ang-(1-7), retaining the critical interactions with TYR91, TYR95, PHE112, TYR248, and HIS263 (Figure 3E). However, unlike Ang-(1-7), it lacked a pronounced charged interaction and instead relied on an extended network of backbone-mediated hydrogen bonds with GLY259, THR270, ILE84, and LEU80 (Figure 3F, Table 1). These interactions indicate a more elongated orientation within the binding groove. Hydrophobic contributions from LEU87, LEU97, LEU266, and CYS83 provided additional but comparatively moderate stabilization relative to dark peptide2 (Figure 3F, Table 1).

Comparative analysis highlights TYR91, TYR95, and PHE112 as a conserved recognition triad engaged by all three ligands. Among the peptides tested, dark peptide2 exhibited the most extensive and diverse interaction network, combining hallmark Ang-(1-7) features with additional electrostatic, π-sulfur, and hydrophobic contacts (Table 1). These findings position dark peptide 2 as the most promising putative Ang-(1-7)-like candidate based on its combined docking interaction profile and binding free-energy estimate. Additional quantitative support for receptor-peptide binding was obtained using molecular mechanics Poisson-Boltzmann surface area (MM-PBSA) binding free-energy analysis. The total binding free energy was favorable for all three complexes, with the most favorable value observed for the MAS-dark peptide 2 complex (-27.03 ± 3.61 kJ/mol), followed by MAS-Ang-(1-7) (-14.62 ± 2.84 kJ/mol) and MAS-dark peptide 1 (-13.15 ± 3.31 kJ/mol). These results provide additional energetic support for stable interaction of the candidate peptides with the MAS receptor and complement the docking scores and residue-level interaction profiles presented in Table 1.

### 3.4. Molecular dynamics simulation of docked complexes

**Figure 4:**
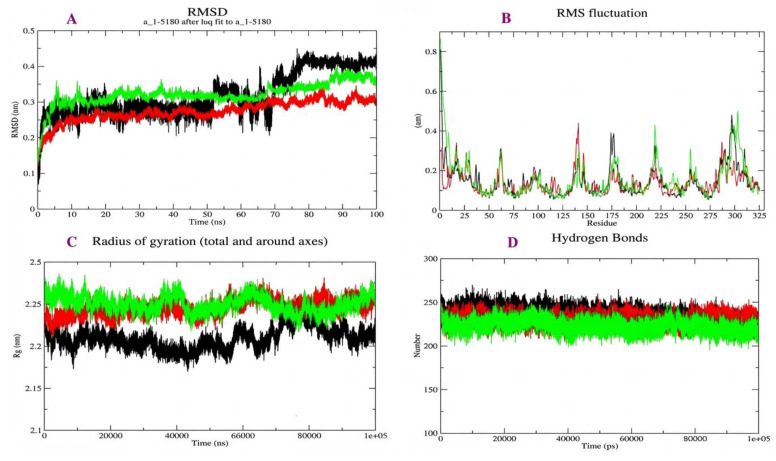
** Molecular dynamics simulation analysis of MAS receptor complexes.** (A) Root mean square deviation (RMSD) profiles showing the overall structural stability of the MAS receptor complexes during 100 ns simulations. (B) Root mean square fluctuation (RMSF) profiles showing residue-level flexibility. (C) Radius of gyration (Rg) analysis showing compactness of the receptor-peptide complexes. (D) Hydrogen-bond analysis showing the stability of receptor-ligand interactions during simulation. Different colors of peak in the graph are as follows Ang-(1-7)-Black, dark peptide 1- Red, dark peptide 2 - Green.

To assess the dynamic stability and conformational behavior of the ligand-receptor complexes, 100-ns MD simulations were performed for MAS-Ang-(1-7), MAS-dark peptide 1, and MAS-dark peptide 2 systems. The RMSD analysis (Figure 4A) showed that all complexes reached equilibrium after approximately 80 ns, maintaining backbone fluctuations within 0.25-0.5 nm. Specifically, the average RMSD values were 0.42 nm for MAS-Ang-(1-7), 0.26 nm for MAS-dark peptide 1, and 0.35 nm for MAS-dark peptide 2 during the final 20 ns of the simulation. The MAS receptor bound to Ang-(1-7) and dark peptide 2 displayed similar stabilization patterns, whereas the MAS-dark peptide 1 complex exhibited minor residual fluctuations beyond 80 ns. Despite its favorable docking score, dark peptide 1 induced slightly reduced structural stability compared with Ang-(1-7) and dark peptide 2, indicating that the latter peptide better mimics the natural ligand in maintaining receptor conformation.

The RMSF profiles (Figure 4B) revealed that loop and terminal regions were more flexible, while residues within the active site—Phe85, Tyr95, Leu97, and His263, remained comparatively rigid across all complexes. This suggests that ligand binding stabilizes the catalytic pocket. The Rg analysis (Figure 4C) demonstrated consistent structural compactness for MAS when complexed with Ang-(1-7) or dark peptide 2, whereas interaction with dark peptide 1 caused slight expansion, reflecting lower compactness and conformational stability.

The hydrogen-bond analysis (Figure 4D) showed that all three complexes maintained a nearly constant number of hydrogen bonds throughout the simulation, confirming that the receptor-ligand interfaces remained stable with minimal fluctuations. Collectively, these MD results indicate that ligand binding enhances the overall stability of the MAS receptor. Both dark peptides engaged the receptor in a manner similar to Ang-(1-7); however, dark peptide 2 demonstrated superior structural compatibility and persistence within the binding pocket, supporting its potential for experimental validation and in-vitro studies.

## 4. Discussion

Despite significant advances in molecular biology and pharmacology, the drug discovery pipeline continues to face major challenges, including high development costs, long timelines, and frequent translational failure. The number of new chemical entities reaching clinical application has declined steadily, largely due to challenges in target validation, translational inefficiency between animal models and human physiology, and escalating clinical-trial costs 30,31. These limitations have encouraged the exploration of alternative sources of therapeutic molecules and the use of computational strategies to accelerate early-stage discovery. One emerging area of interest is the “genomic dark matter,” which includes non-coding and poorly annotated genomic regions that may harbor previously unrecognized functional elements 32-34.

In the present study, we explored intergenic regions of the *Saccharomyces cerevisiae* genome as a potential source of short bioactive peptides. This work builds upon our earlier studies demonstrating that functional proteins can be synthesized from non-coding regions of the *Escherichia coli* genome, including one intergenic-sequence-derived protein that inhibited bacterial cell growth 35. Subsequent studies by our group also predicted functional and structural correlates for proteins encoded by evolutionarily retired pseudogenes 36. This approach is further supported by growing evidence from ribosome profiling and proteogenomic studies showing that non-canonical open reading frames can be transcribed and translated into short peptides or microproteins 2-6. Although intergenic regions have traditionally been considered non-coding, recent studies indicate that some unannotated sequences may contribute to regulatory, metabolic, or signaling processes. Therefore, mining these regions may expand the searchable sequence space for peptide-based drug discovery.

The identified dark peptides exhibited both sequence and structural similarity to Ang-(1-7), supporting the possibility that short peptide motifs derived from non-coding genomic regions can adopt conformations resembling known bioactive ligands. However, because Ang-(1-7) is a short heptapeptide, sequence similarity values should be interpreted with caution, as partial matches may occur by chance in short-sequence comparisons. Therefore, sequence similarity was used only as an initial screening criterion rather than as definitive evidence of functional equivalence. Candidate prioritization and evaluation were instead based on multiple converging computational features, including ligand-like length, conservation of relevant physicochemical properties, structural similarity to Ang-(1-7), compatibility with the predicted MAS receptor-binding pocket, conservation of key receptor-contact interactions, and stability during molecular dynamics simulation. Moreover, the present study does not provide direct experimental evidence that these specific intergenic sequences are naturally transcribed, translated, or processed into stable peptides in *S. cerevisiae*. Future RNA sequencing, ribosome profiling, and targeted proteomic analyses will therefore be required to confirm endogenous expression and peptide production.

The MAS receptor is a class A GPCR involved in the protective arm of the renin-angiotensin system 37-39. In this study, the modeled MAS receptor displayed the expected seven-transmembrane architecture, and docking analyses suggested that the intergenic peptides could occupy the predicted ligand-binding region. The conservation of interactions with residues implicated in Ang-(1-7) recognition supports the possibility that these peptides may mimic some binding features of the native ligand. Among the candidates, dark peptide 2 showed the most favorable overall computational profile, including strong structural resemblance, extensive receptor-contact interactions, favorable MM-PBSA binding free energy, and stable receptor engagement during simulation.

Molecular dynamics simulations further supported the stability of the receptor-peptide complexes in a membrane environment. However, stable docking poses and molecular dynamics trajectories do not establish MAS receptor activation or downstream signaling. This distinction is particularly important for GPCRs, where ligand binding does not necessarily translate into agonistic or antagonistic activity, G protein coupling, β-arrestin recruitment, or pathway-specific signaling. Therefore, the identified peptides should be considered putative MAS-binding candidates rather than confirmed functional activators. Ang-(1-7)-MAS signaling has been associated with several downstream pathways, including Gq/phospholipase C-mediated calcium signaling, phospholipase A2 activation, PI3K/Akt signaling, nitric oxide production, and β-arrestin-related responses. Future studies should evaluate the synthesized peptides in MAS-expressing cell systems to determine whether they functionally mimic Ang-(1-7) and whether they upregulate or downregulate PLC, PLA2, PI3K/Akt, nitric oxide, or β-arrestin signaling.

Peptide-based therapeutics are increasingly attractive because of their specificity, potency, and synthetic accessibility 40-41. Nevertheless, short peptides often face limitations such as poor proteolytic stability, rapid clearance, low oral bioavailability, and limited membrane permeability 40,43. The intergenic peptides identified here should therefore be considered preliminary lead-like candidates that may require chemical optimization, stability enhancement, and functional validation before any therapeutic relevance can be established.

Experimental validation remains essential to confirm the biological activity and translational relevance of these candidates. Future studies should synthesize the candidate peptides and evaluate MAS receptor binding using ligand-binding or competition assays. In vitro stability assays and subsequent in vivo studies will also be required to determine pharmacological relevance.

Overall, this study provides a computational framework for identifying short ligand-like peptides from intergenic genomic regions. While experimental validation is essential, the findings support the broader concept that non-coding genomic regions may serve as an underexplored reservoir of peptide sequences with potential relevance to receptor-targeted drug discovery 6,44.

## 5. Conclusion

This study demonstrates that non-coding regions of the Saccharomyces cerevisiae genome encode short peptides with potential therapeutic relevance. Using an integrated computational pipeline, we identified intergenic peptides that exhibit strong structural similarity to Ang-(1-7) and stable binding to the MAS receptor. Molecular docking and dynamics simulations revealed conserved interaction patterns and sustained receptor engagement, with dark peptide 2 emerging as the most promising functional mimic of the native ligand. These findings highlight genomic dark matter as a previously unexplored source of bioactive peptide ligands. However, because the present study is based entirely on computational analyses, experimental validation is required before biological activity or therapeutic potential can be confirmed. Future studies, including peptide synthesis, receptor-binding assays, MAS-dependent signaling assays, and peptide stability analysis, will be necessary to validate the functional relevance of these candidates.

## Supplementary Material

Supplementary figure.

## Figures and Tables

**Table 1:** Comparative Summary of MAS Receptor Interactions for Ang-(1-7), dark peptide1, and dark peptide2

Peptide	ZDOCK Score	ZRank Score	MM-PBSA ΔTOTAL Binding Free Energy (kJ/mol)	Electrostatic / Charged Interactions	All Hydrogen Bonds	Aromatic Interactions	Hydrophobic & Aliphatic Contacts	Total contacts
Ang-1-7	11.08	-68.77	-14.62 ± 2.84	GLU167 (salt bridge), ARG245 (H-bond)	TYR91, TYR95, TYR248, ARG245	PHE112, HIS262, HIS263, TYR248	LEU97	11
Dark peptide1	10.98	-95.66	-13.15 ± 3.31	None	TYR91, TYR95, GLY259, THR270, ILE84, LEU80	PHE112, TYR248, HIS263	LEU87, LEU97, LEU266, CYS83	13
Dark peptide2	11.78	-66.34	-27.03 ± 3.61	ASP90 (attractive charge)	TYR91, TYR95, ASN260, HIS263	PHE112 HIS263 CYS83	LEU87, LEU97, LEU108, LEU266, VAL35, ALA28	14
