# Supplementary material for: Computational Discovery of Angiotensin-(1-7)-like Peptides Targeting the MAS Receptor from the Genomic Dark Matter of Saccharomyces cerevisiae
Source: Int J Med Sci. 2026 Jul 1;23(8):2568–77. doi: 10.7150/ijms.135093 (PMC13411471; doi:10.7150/ijms.135093)
Supplement: Supplementary file 1 — Supplementary figure. [file ijmsv23p2568s1.pdf]

PROCHECK

Ramachandran Plot

Mass receptor

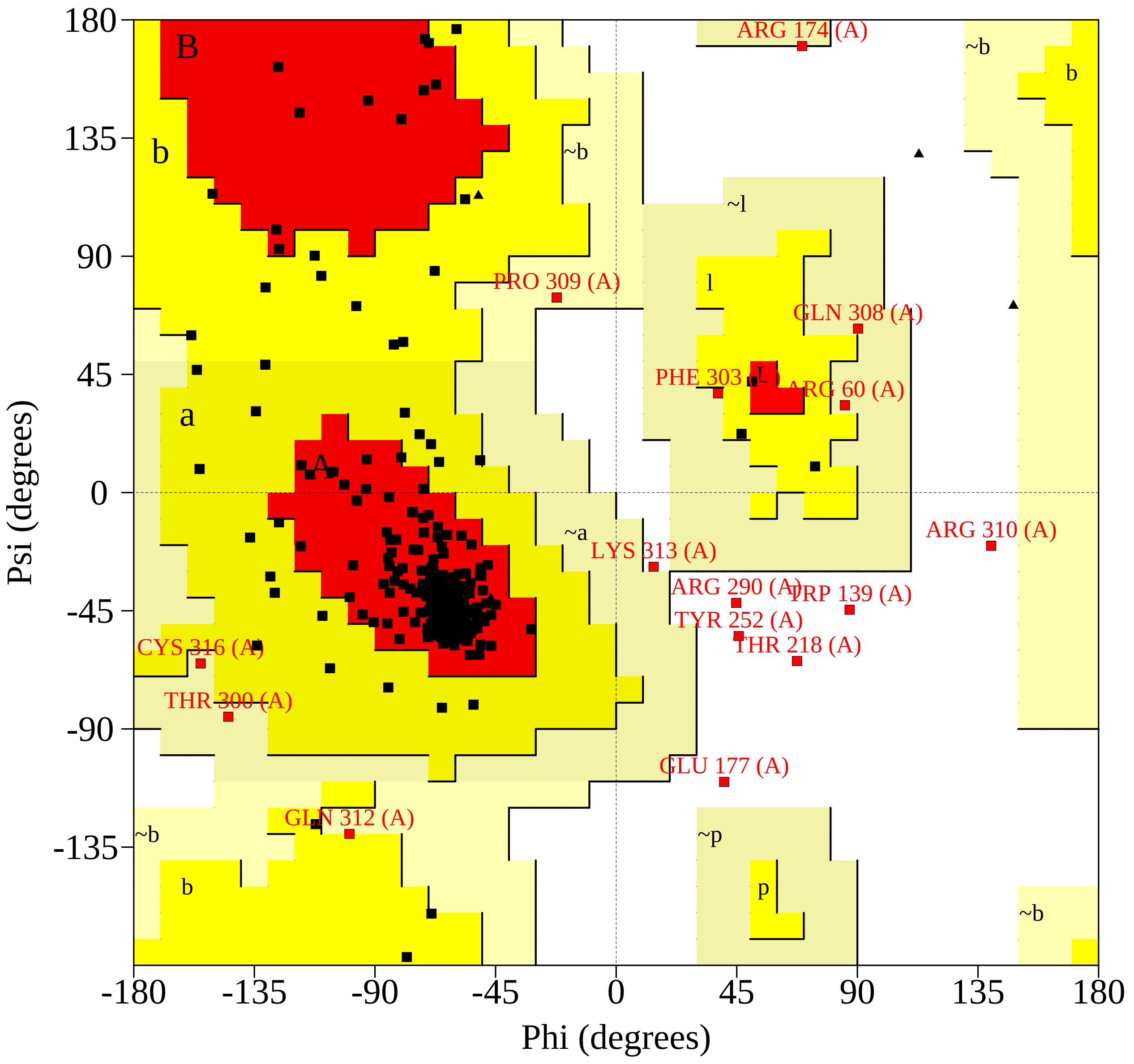

Plot statistics

Residues in most favoured regions [A,B,L]	253	83.5%
Residues in additional allowed regions [a,b,l,p]	36	11.9%
Residues in generously allowed regions [~a,~b,~l,~p]	7	2.3%
Residues in disallowed regions	7	2.3%
	----	------
Number of non-glycine and non-proline residues	303	100.0%
Number of end-residues (excl. Gly and Pro)	2	
Number of glycine residues (shown as triangles)	10	
Number of proline residues	10	

Total number of residues	325	

Based on an analysis of 118 structures of resolution of at least 2.0 Angstroms and R-factor no greater than 20%, a good quality model would be expected to have over 90% in the most favoured regions.
